# Business Cycle and Public Health: The Moderating Role of Health Education and Digital Economy

**DOI:** 10.3389/fpubh.2021.793404

**Published:** 2022-01-11

**Authors:** Xing Zhang, Yingying Xu

**Affiliations:** ^1^School of Finance, Renmin University of China, Beijing, China; ^2^School of Economics and Management, University of Science and Technology Beijing, Beijing, China

**Keywords:** business cycle, public health, health education, digital economy, moderating effect

## Abstract

The cyclicality of public health in the emerging market is underexplored in existing literature. In this study, we used a fixed effect model and provincial data to document how public health varies with the business cycle in China over the period of 2010–2019. The estimated results showed that the business cycle is negatively correlated with the mortality of infectious disease, a proxy variable of public health, thus indicating that public health exhibits a countercyclical pattern in China. Furthermore, we investigated the potential moderating role of public health education and digital economy development in the relationship between business cycle and public health. Our findings suggested that public health education and digital economy development can mitigate the damage of economic conditions on public health in China. Health education helps the public obtain more professional knowledge about diseases and then induces effective preventions. Compared with traditional economic growth, digital economy development can avoid environmental pollution which affects public health. Also, it ensures that state-of-the-art medical services are available for the public through e-health. In addition, digitalization assures that remote working is practicable and reduces close contact during epidemics such as COVID-19. The conclusions stand when subjected to several endogeneity and robustness checks. Therefore, the paper implies that these improvements in public health education and digitalization can help the government in promoting public health.

## Introduction

The worldwide public health emergency, i.e., COVID-19, has caused many infections and deaths, which has revealed the importance of public health preparedness and raised wide concern about public health throughout the world. Given potential outbreaks and evolution of COVID-19 and other pandemics, public health continues to be the focus of attention in the post-COVID-19 period. The spread of infectious diseases (e.g., COVID-19) can destroy public health and generate negative spillovers to other sectors (1). A public health crisis is widely viewed as a “top global economic risk,” thus creating worldwide uncertainty, and generating enormous economic impacts (2, 3). In turn, an economic crisis also affects public health. The incidence of infectious diseases such as influenza, HIV, etc., increased after the 2008 global financial crisis. However, government budgets on public health services declined during the 2009–2010 European debt crisis. Therefore, during the economic downturn caused by COVID-19, the relationship between business cycle and health issues has once again become of interest to academics (4).

Public health is closely related to economic conditions in emerging markets. As the most typical emerging country, China has achieved rapid development since the economic reform in 1978. However, part of the economy benefits is obtained at the cost of the environment, thus causing some people to be infected by various pollution-generated diseases. Besides, society becomes aggressive and competitive because of the rapid economic growth with many people exhibiting impetuous, indifferent, stressful, or other negative psychology behaviors, which are harmful to public health. In fact, China has made great efforts to improve public health after realizing those issues, including public health campaigns, economic structure transformations, new energy vehicle promotions, and carbon neutrality targets by 2060 (5–7). The prevalence of infectious disease has been significantly reduced by improved sanitation, cleaner water, fresh air, safer food, and the effective infectious disease prevention and control system. Moreover, two documents of *the Healthy China 2030 Plan* and *Guideline on the Development of Internet plus Healthcare* were separately released in 2016 and 2018, which enhanced public health advocation and enriched public health services.

Health education is associated with good health through disseminating necessary health information and fostering motivation and skills for adopting healthy choices (8). Those who are well-educated on health literacy will make healthier choices owing to their better health awareness and professional knowledge of disease prevention. These healthy choices and behaviors may positively impact health outcomes. The prospect of effective public health education in reducing mortality during COVID-19 has been examined by a mathematical model developed by Iboi et al. (9). Because of improved medical understanding of COVID-19, educated individuals and the general community have been wearing facial masks, maintaining social distancing, and avoiding close contact, which has resulted in lower transmission rates and lower incidence levels (10). Sometimes, public health education requires a break from cultural practices which may contribute to the spread of the disease, for example, the U.S. citizens who changed their mind about wearing a facial mask to prevent COVID-19. Public health education or professional training are lacking in China due to inadequate vocational funding and supplies, which greatly limits the development and improvement of public health capabilities (11). According to a report of the Association of Schools and Programs of Public Health (ASPPH), in 2020, there were 77 universities offering public health programs, and only 46 universities were authorized to enroll Master of Public Health students for which about 6,500 students can be cultivated per year. China has specially created a certification program to conduct the public health medical practitioner test for public health students, but only 60,000 students can pass the test each year (12). In general, there is a huge shortage of public health training. Moreover, in the past decades, learning how to deal with a public health crisis related to pandemic outbreaks such as SARS, H1N1, and COVID-19 is still considered non-essential training (13). The lack of medical resources and healthcare workers at the beginning of COVID-19 indicated that health preparedness is not inadequate in China, which is due to the lack of regular public health emergency training in normal times. In brief, public health education in China needs more attention. Thus, this paper explores the role of health education in contemporary health promotion and attempts to promote public attention to the crucial role of health education in public health promotion.

Meanwhile, one universal phenomenon is that digitalization has facilitated various social (sub-)systems, including politics, economy, finance, governance, health, etc. Many digital tools have been used in response to the COVID-19 epidemic, including Internet of Things (IoT), AI, 5G etc. (14). One stand-out area in this regard is apps. Many mobile apps were used for different purposes, playing a crucial role in remediating the COVID-19 outbreak (15). For example, automated digital contact tracing was used for participatory surveillance, Zoom meetings were used for remote working or learning, social media was used to target communication or public health promotion. In 2020, there were over 3.6 billion social media users worldwide (16). In China, the number was around 926.8 million (17). Among them, WeChat was by far the most popular social media app in China, which was used to release official government messages and distribute important public health information.

Healthcare is currently experiencing a digital transformation. Mobile-health (m-health) and electronic-health (e-health) are the most rapidly developing applications of health in recent years. M-health denotes public health practices supported by more mobile devices. M-health has the advantages of being simple, low cost, and user-friendly. It also helps to ensure the speed and accuracy of healthcare delivery. E-health includes health services provided by information and communication technologies (ICTs) (18). ICTs plays a vital role in improving public health as they can bridge the information divide between health professionals and individuals by providing communities with an efficient tool to access, communicate, and store information. It is recognized that ICTs will be the key resources to strengthen the future preparedness for infectious diseases and achieve the challenge of public health in the twenty-first century (19, 20). Moreover, the COVID-19 pandemic has accelerated the use of e-health. E-health may be more preferred by the public in the post-COVID-19 phase. The future of public health will become increasingly digital (19). Many countries have taken digital technologies consideration into their public health plans to strengthen their health system (21). In early 2020, the beginning of the pandemic, the Chinese government emphasized the significance of e-health when the National Health Commission issued the report *Opinions on E-Health Consultation and Service in the Pandemic Period*. After this the demand for online e-health dramatically increased, and the health consultations in e-health apps increased more than 20 times compared to the previous year. Ping An Good Doctor, the most popular e-health app in China, had 67.3 million users monthly during the pandemic. For another e-health app led by Alibaba, Ali health, the daily average consultations were 100 patients per doctor. Informationization is the essence of digital economy. The Chinese government has been greatly promoting digital transformation to develop digital economy in recent years. Therefore, we attempt to examine whether public health benefits from digital economy development in the Chinese context.

Since pandemics like COVID-19 may continue to exist for many years, the importance of making these future-proofing efforts cannot be overstated (13). It is necessary to investigate how to better respond to the next pandemic. This paper aims to show the vital role of public health education and digital development in decreasing the mortality of infectious disease. To the best of our knowledge, there is no study on the moderating role of public health education and digital economy in the relationship between business cycle and public health. Our contributions to the literature are as follows. First, we provide estimates for the cyclicality of public health by examining the impact of business cycle on the mortality of infectious disease in China, which has not been revealed in existing literature. Most previous studies have discussed how business cycle is correlated to mortality. This paper will enrich the study on business cycle and public health, moreover, it will raise the academic attention on infectious disease in the period of post-COVID-19. Second, we indicate how public health education and digital economy may influence the impact of business cycle on public health. Although the significance of health education has been theoretically and practically emphasized, the empirical evidence is still rare. Our study fills this research gap to some extent. In addition, it may accelerate the application of advanced technology in medical services by evidencing the role of digitalization in improving public health, which in turn helps contribute to digital economy development, increasing social welfare.

This study is constructed as follows. Section Literature Review and Research Hypotheses reviews previous literature and proposes research hypotheses. Section Data and Methodology introduces the data and empirical models employed in this study. Results and discussion are presented in section Empirical Results and Discussion, and section Robustness Checks conducts robustness checks. The section Conclusions concludes this study.

## Literature Review and Research Hypotheses

There is wide interest among academics and policymakers in understanding the relationship between business cycle and public health, with a particular focus on population mortality (22). However, the impact of business cycle on mortality is somewhat controversial in previous empirical investigations (23). Some literature addressed pro-cyclical effects of business cycle on mortality in Greece, Finland, Iceland (24), Spain (22), Canada (25), England and Wales (26, 27), the United States (28), Japan (29), and ASEAN countries (30), thus showing that economic recession promotes public health and decreases mortality. By contrast, some other studies asserted a procyclical pattern of mortality, indicating that economic recession was not good for public health (31, 32). There are some other studies that found no significant effect of business cycle on mortality (33) or a changing relationship between business cycle and mortality across various time periods (28, 31, 34). For instance, in the past decades, the United States and South Korea both experienced a dramatic change from counter-cyclicality to pro-cyclicality of public health (35, 36). As for the theoretical mechanism of how macroeconomic conditions affect public health, very little is yet known about the precise mechanisms. A potential channel is that a recession reduces the opportunity cost and encourages health behaviors.

Besides the investigation on how business cycle affects population mortality, a related strand of the literature has focused on exploring the impacts on morbidity or mortality of infectious diseases. Hunter et al. (37) reported a non-linear relationship between business cycle (measured by GDP per capita and unemployment rate) and infectious disease mortality. However, the concrete mechanisms behind the relationship between business cycle and public health remain poorly understood. In the context of COVID-19, Goutte et al. (38) and Shahbazi and Khazaei (39) both found that morbidity and mortality of COVID-19 are higher with better socioeconomic status, thus indicating a negative impact of business cycle on public health. According to Gonzalez and Quast (40), infectious disease is countercyclical in less developed areas but procyclical in better developed areas. The relationship between public health and business cycle varies by the level of development. Previous studies have focused on the relationship between business cycle and health outcomes in developed countries, and little is known about emerging market economies. China exhibits almost all the notable features that characterize emerging market countries (41). Moreover, China is a major contributor to the worldwide infectious disease burden because of its population size. Thus, this paper aims to provide evidence on how public health related is to business cycle in an emerging country, China, by exploring the correlation between business cycle and infectious diseases mortality (IDM). We thus propose the following hypothesis.

### Hypothesis 1

Business cycle is significantly correlated to public health in China.

The effects of public health education on the evolution of diseases have been investigated worldwide since the outbreak of several infectious diseases, such as HIV (42–44) and Ebola (10, 45). The studies on the role of health education in promoting public health have reached an agreement (46). Some recent studies suggested that public health education can provide the general population with a better understanding of the disease and help them take efficient measures to prevent the disease (42, 43). Especially during a public health emergency, public involvement is the crucial power to alter the pandemic and curtail the spread of disease. Target education through practical health information to the public is the essential means to get the public involved.

Moreover, the channels of education have expanded from newspapers, lectures, and posters to digital tools, such as text messages, mobile apps, and websites. Among them, social media is most accessed by the public and plays the mostly increasing role in health education due to its interactivity. Social media facilitates greater information sharing and bridges the communications between the public and professional medical knowledge providers. Health promotion-related social media websites are currently popular all around the world (47). The use of social media in public health education and promotion is also increasing in China. According to the survey conducted by Shen (48), COVID-19 has aroused the attention of public health education in China. There are nearly three quarters of residents who are aware of public health and support the promotion of public health education and training, whereas that proportion was only about 20% before the outbreak of COVID-19. Thus, we propose the following hypothesis.

### Hypothesis 2

Public health education moderates the negative impact of business cycle on public health.

Advances in technology are central to digital economy development. Digital technology has greatly supported public health prevention during the COVID-19 pandemic through the adoption of e-health. The intersection of e-health in public health is viewed as a “beautiful marriage” (49). It is important to note that most of the previous research mainly focused on explaining how digital technology is applied to tackle COVID-19. Budd et al. (19) noted that digital technologies contributed to the response to COVID-19 by offering tools for population surveillance, case identification, contact tracing, etc. Moreover, digital technologies have the advantage of real-time data to support containment measures for COVID-19 (50). Keesara et al. (51) pointed out the usefulness of digital tools in strengthening the public health care system. Ting (14) summarized the potential use of four inter-related digital technologies in public health strategies of COVID-19. Among them, IoT provides a platform for monitoring public health. Big data analysis provides modeling results for guiding the policymakers to take more effective measures. AI helps to enhance the detection of COVID-19. The use of blockchain in hospitals can ensure timely delivery of medications with accurate tracking. Besides, there are some other digital technologies used to fight the pandemic. For example, Singh et al. (52) explored the use of three-dimensional printing in public health. Wirth et al. (15) identified the role of citizen-centered mobile health apps in supporting public health, for instance, collecting individual-level data for better infectious disease management. In fact, before the outbreak of COVID-19, the role of digital technologies in promoting public health was recognized by many studies. George et al. (53) documented how those technologies can accelerate the application of forecasting in practice, improve public health management, and mitigate the economic impact of outbreaks. Wójcik et al. (54) pointed out the role of participatory surveillance systems in promoting information communication between the general population and public health professionals.

From the discussion of previous studies, digital technology is useful in supporting and enhancing public health management. Thus, we propose the following hypothesis.

### Hypothesis 3

Digital economy development moderates the negative impact of business cycle on public health.

## Data and Methodology

### Data

#### Dependent Variables

The unemployment rate is a preferred measure of business cycle in developed or high-income countries. However, it might be a poor measure of business cycle in developing countries (55). In the context of the emerging market of China, we used the real annual GDP growth rate (*rgdpg*) as a proxy for business cycle as the dependent variable according to previous studies (56, 57).

#### Independent Variable

Infectious disease is the leading threat on public health, and the public health department is mainly responsible for preventing epidemics and the spread of disease (58). The Chinese government has established a complete system to prevent infectious diseases in the past decades. However, infectious diseases have always been a major public health threat. Thus, we used infectious disease mortality (*idm*) to measure public health as the independent variable.

Furthermore, we considered two moderating variables, the first one is public health education, which is measured by the number of public health education activity (*edu_act*). We also adopted an alternative proxy variable of health education, which is measured by person-time of health education training (*edu_train*) to conduct a robustness check. The second moderating variable is digital economy. The digital economy refers to all activities that can be undertaken using ICT tools. The Asian Development Bank Institute defines the digital economy as the usage of the Internet, cloud computing, big data, fintech, and other new digital technologies to collect, store, analyze, and share information digitally and transform social interactions (59). We used China's digital economy development index (*dedi*) as the proxy variable and selected the Digital Financial Inclusion Index of China (*dfiic*) in the robustness test.

### Control Variables

There are four groups of control variables in the province level, i.e., the density of medical resource which is measured by the number of medical institutions (*mid*), medical beds (*beds*), and licensed doctors (*doctors*) per 1,000 people to control the effect of medical resource, the proportion of secondary industry to total GDP (*s_ind*) to control the effect of economic structure, the proportion of the population aged over 65 to the total population (*aging*) to control the effect of aging degree of the population, and the proportion of urban population to total population (*ur*) to control the effect of urbanization level. The definitions of the variables are listed in Table 1.

**Table 1:** Definition of variables.

**Types**	**Variables**	**Indicators**	**Symbols**	**Definitions**	**Source**
Dependent variables	Public health	infectious disease mortality	*idm*	Number of infected per 10,000,000 people	China National Health Statistical Yearbook
Independent variables	Business cycle	Real GDP growth rate	*rgdpg*	The real annual growth rate of GDP	
	Health education	Public health education activity	*edu_act*	Number of public health education activity/Population	
		Health education training	*edu_train*	Person-time of health education training/Population	
	Digital level	China digital economy development index	*dedi*	China digital economy development index, released by CCID Consulting Co.	CCID Consulting
		Digital Financial Inclusion Index of China	*dfiic*	The Peking University Digital Financial Inclusion Index of China, calculated by Guo et al. (60)	http://idf.pku.edu.cn
Control variables	Density of medical resource	Medical institutions	*mid*	Number of medical institutions per 1,000 people	China National Health Statistical Yearbook
		Medical beds	*beds*	Number of medical beds per 1,000 people	
		Licensed (assistant) doctor	*doctors*	Number of licensed (assistant) doctors per 1,000 people	
	Economic structure	Secondary industry ratio	*s_ind*	The proportion of GDP in the secondary industry to total GDP	
	Population characteristics	Aging ratio	*aging*	The proportion of the population aged over 65 to the total population	
	Urbanization level	Urbanization rate	*ur*	The proportion of the urban population to the total population	

Due to the lack of data in Tibet province, this paper used panel data of 30 provinces in China during the decade of 2010–2019 to empirically test the relationship between business cycle and public health. Data were obtained from the China National Health Statistical Yearbook. However, for two digital indicators, *dedi* was only available during 2012–2019 released by CCID Consulting, and *dfiic* was only available during 2011–2019 updated by the Institute of Digital Finance Peking University. Finally, to eliminate the influence of outliers, 1% winsorization was processed.

### Methodology

Based on the hypotheses proposed above and referring to the related literature (31, 34), the baseline econometric model is established as follows,

(1) $$\begin{array}{r} Health_{i,t}=\alpha +\beta {Bus\text{\_}cycle}_{i,t}+\gamma Controls+\mu_{i}+\tau_{t}+\varepsilon_{it} \end{array}$$

where *Health*_*i,t*_ represents the public health of province *i* in period *t*. We use *idm* as the proxy. *Bus*_*cycle*_*i,t*_ represents the business cycle of province *i* in period *t*. We use *rgdpg* as the proxy. *Controls* represents control variables, including *mid, beds, doctors, s_ind, aging*, and *ur*. μ_*i*_ and τ_*t*_ separately control the fixed-effects for province and year. ε_*it*_ is the random interference term.

Furthermore, regarding the theoretical analysis above, we explored the moderating role of health education (*Edu*) and digital economy development (*Digi*) in the association between business cycle and public health. We separately introduced the cross-product of *Bus*_*cycle*_*i,t*_ and *Edu*_*i,t*_, *Bus*_*cycle*_*i,t*_ × *Edu*_*i,t*_, the cross-product of *Bus*_*cycle*_*i,t*_ and *Digi*_*i,t*_, *Bus*_*cycle*_*i,t*_ × *Digi*_*i,t*_ into Equation (1). Then the model is constructed as:

(2) $$\begin{array}{l} Health_{i,t}=\alpha +\beta_{1}{Bus\text{\_}cycle}_{i,t}+\beta_{2}Edu_{i,t}\\ \;+\beta_{3}{Bus\text{\_}cycle}_{i,t}\times Edu_{i,t}+\gamma Controls+\varepsilon_{it} \end{array}$$

(3) $$\begin{array}{l} Health_{i,t}=\alpha +\beta_{1}{Bus\text{\_}cycle}_{i,t}+\beta_{2}Digi_{i,t}\\ \;+\beta_{3}{Bus\text{\_}cycle}_{i,t}\times Digi_{i,t}+\gamma Controls+\varepsilon_{it} \end{array}$$

## Empirical Results and Discussion

### Descriptive Statistics

Descriptive statistics are summarized in Table 2. The average mortality of infectious disease in 30 provinces during 2010–2019 was 0.2457 per 10,000,000 people. The infectious disease mortality (*idm*) varied significantly during the sample period with the maximum being 0.6217, the minimum being 0.1131, and a standard derivation of 0.0954. The variable of business cycle, *rgdpg*, had similar descriptive statistical characteristics to infectious disease mortality. Health education and digital economy in most of provinces were still at a low level although having improved in recent years. More than half of the samples had less health education and lower digital economy than the average level as the mean value of *edu_act* and *dedi* were both larger than median value (*p50*). Similar with the statistics characteristics of health education and digital economy, the three variables of density of medical resources, *mid, beds*, and *doctors*, suggested most provinces had inadequate medical resources in China during the sample years.

**Table 2:** Descriptive statistics.

**Variable**	***N***	**Mean**	**sd**	**Min**	**p25**	**p50**	**p75**	**Max**
*idm*	300	0.2457	0.0954	0.1131	0.1874	0.2309	0.2754	0.6217
*rgdpg*	300	0.1044	0.0687	−0.2555	0.0740	0.1037	0.1436	0.2866
*edu_act*	300	0.7747	1.2087	0.0008	0.1795	0.3981	0.9153	11.4156
*edu_train*	300	15.8300	24.8578	0.0717	3.6647	8.0184	17.2659	223.8735
*dedi*	240	0.2921	0.1690	0.0237	0.1720	0.2629	0.3666	0.8109
*dfiic*	270	5.1331	0.6457	1.9110	4.9043	5.2467	5.6111	6.0866
*mid*	300	7.0956	2.3528	2.0400	5.4381	7.2322	8.8891	11.3899
*beds*	300	5.0351	1.1105	2.7250	4.2250	4.9500	5.8450	7.4300
*doctors*	300	2.2931	0.6221	1.2500	1.9000	2.2550	2.5100	5.0700
*s_ind*	300	0.4414	0.0874	0.1620	0.3960	0.4595	0.5030	0.5900
*ur*	300	0.5709	0.1263	0.3381	0.4865	0.5567	0.6235	0.9415
*aging*	300	0.1014	0.0218	0.0547	0.0855	0.0985	0.1154	0.1626

*N is the sample observation; mean and sd denote the average value and standard derivation of the variable, respectively; min and max denote the minimum and maximum of the variable, respectively; p25, p50, and p75 represent the 25, 50, and 75% percentile of the variable, respectively. idm represents infectious disease mortality, rgdpg represents real GDP growth rate, edu_act represents public health education activity, edu_train represents health education training, dedi represents China digital economy development index, dfiic represents digital financial inclusion index of China, mid represents medical institutions density, beds represents medical beds density, doctors represents licensed (assistant) doctor density, s_ind represents the secondary industry ratio, ur represents urbanization rate, aging represents aging ratio*.

**Figure 1:**
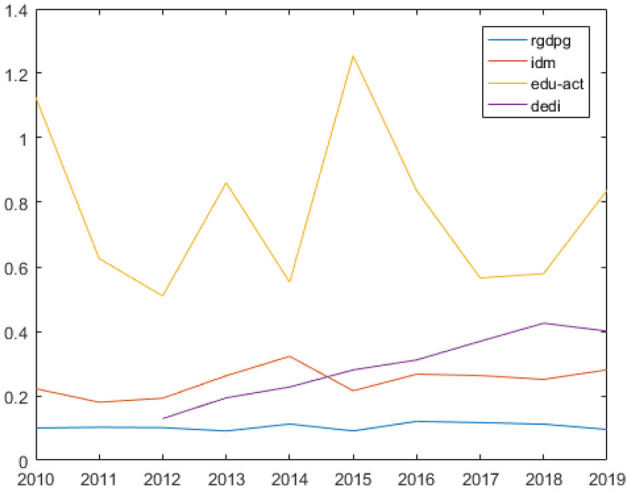
Business cycle, public health, health education, and digital economy in China during 2010–2019.

As can be seen from Figure 1, *idm* increased with the *increase* of *rgdpg* and decreased with the decrease of *rgdpg* in most of the sample years. This indicates that public health was counter-cyclical with business cycle in China in the past 10 years. However, the trend of *edu_act* was opposite to that of *rgdpg*. It means that less public health education was provided compared with the higher GDP growth rate. The digital economy was significantly developed in China during 2012–2019.

### The Effect of Business Cycle on Public Health

We conducted the Hausman test, and a fixed effect model was suggested rather than random effect as the test results reject the null hypothesis. According to a previous study (34), we examined the impact of business cycle on public health by employing the fixed effects model to estimate Equation (1). The results are summarized in column (1) of Table 3. The coefficients of *rgdpg* to *idm* were significantly positive at the 1% level, which can be interpreted as a harm of public health with higher economic growth. Public health exhibits a countercyclical pattern in China. One possible causation is that China has achieved great economic growth in the past decades at the cost of environmental pollution, air pollution, water pollution, and waste pollution, which causes the spread of infectious disease and endangers public health. Although the central government has paid much attention to public health issues since the economy has improved, many local decision makers who are driven by economic interests still aim at obtaining short-term benefits from projects and ignore their responsibility on public health.

**Table 3:** Empirical results.

	**(1)**	**(2)**	**(3)**
	***idm***	***idm***	***idm***
*rgdpg*	0.272^***^	0.391^***^	0.614^***^
	(3.93)	−4.71	(5.89)
*edu_act*		0.0016	
		(0.47)	
*rgdpg_act*		−0.227^***^	
		(−5.32)	
*dedi*			−0.0212
			(−0.12)
*rgdpg_dedi*			−0.4680^**^
			(−2.43)
*mid*	−0.0001	0.0015	0.0100
	(−0.01)	(0.10)	(0.51)
*beds*	0.0117	0.0086	0.0380
	(1.17)	(0.82)	(1.29)
*doctors*	−0.0306	−0.0298	−0.0256
	(−1.49)	(−1.41)	(−1.19)
*s_ind*	−0.0382	−0.0060	−0.251^***^
	(−0.84)	(−0.11)	(−5.54)
*ur*	0.0218	0.0664	−0.625
	(0.10)	(0.29)	(−1.36)
*aging*	1.577^**^	1.594^**^	1.265
	(2.32)	(2.27)	(1.13)
*cons*	0.0735	0.0379	0.347
	(0.59)	(0.30)	(1.48)
*N*	300	300	240
*adj.R-sq*	0.0740	0.110	0.0870

*t statistics in parentheses*

* *p < 0.1,*

** *p < 0.05,*

*** *p < 0.01*.

*idm represents infectious disease mortality, rgdpg represents real GDP growth rate, edu_act represents public health education activity, rgdpg_act is the cross product of rgdpg and edu_act. dedi represents China digital economy development index, rgdpg_dedi is the cross product of rgdpg and dedi, mid represents medical institutions density, beds represents medical beds density, doctors represents licensed (assistant) doctor density, s_ind represents the secondary industry ratio, ur represents urbanization rate, aging represents aging ratio. cons is the constant term, and N is the sample observation*.

Another possible causation is that, due to economic development and convenient transportation, infectious disease has become easier to spread nationwide through travel, which can be seen from the spread of COVID-19. Moreover, the advance of urbanization has increased competition within society, which has increased the psychologically pressure of the public. Suffering from fierce competition and depression for a long time is harmful for health. In turn, mental health tends to decrease during economic slowdown. The possible mechanism is that, during the economic downturn, the public are more willing to take part in health-producing activities due to the decrease of the opportunity cost of time, which is good for their health. Thus, it is important for the public health department to pay more attention to the mental health of the public with the rapid development of economy, in addition to physical health. In summary, the empirical results support hypothesis 1 that business cycle is an economically important risk factor for public health, which is consistent with the finding of Chai et al. (61) that business cycle is negatively correlated with population health.

### The Moderating Role of Public Health Education

We proceeded to explore the moderating role of public health education in affecting the relationship between business cycle and public health using Equation (2). The estimated results are shown in column (2) of Table 2. The coefficient of *rgdpg* maintained significantly positive to *idm*, consistent with the above finding. However, the interaction term of *rgdpg_act* had a negative coefficient to *idm* at the 1% significance level, which was opposite of the sign of the coefficient of *rgdpg*, thus confirming that the moderating effect of health education does exist. It suggests that public health education helps to weaken the negative impact of business cycle on public health in China.

There are two possible reasons. First, the public can obtain more professional knowledge about the disease and will take preventions to avoid being infected. For example, during the COVID-19 pandemic, the public were educated about the transmission pathways of COVID-19 to enable them to protect themselves and stop the spread of the virus by wearing facial masks, social distancing, and avoiding gathering and unnecessary travels, etc. Second, health education is available for more people with the development of digitalization. The health awareness of the general population will continuously increase and will not change with economic fluctuations. A high level of health awareness is essential to keep the public healthy. The results of this study provide an evidence-based reference for health policy decision-making on developing public health education in the future. Our results are consistent with the second hypothesis that health education helps to mitigate the potential deleterious effects of economic growth on public health.

### The Moderating Role of Digital Economy Development

Similarly, we also investigated the moderating role of digital economy development. The estimated results are shown in column (3) of Table 2. The significantly positive coefficient of *rgdpg* further verified hypothesis 1. The coefficient of the interaction term *rgdpg_dedi* to *idm* was significant and opposite to the sign of *rgdpg*, which indicates the moderating effect of digital economy development on the association between business cycle and public health. One potential explanation is that e-health removes the physical barriers that traditionally impede access to healthcare and makes the best medical support and resources available for more people. Especially during the pandemic, digital health has satisfied the healthcare needs of residents whose travel was constrained. In addition, it is the digitalization that assures remote working is practicable, and effectively avoids close contact in the office.

Moreover, digital economy development reduces harmful effects of business cycle on public health as *rgdpg_dedi* had a larger coefficient (−0.468) than that of *rgdpg_act* (−0.227). OECD (62) noticed that digital economy helps to create more jobs. The increased income will promote the public's well-being and further promote their health, as it greatly empowers an individual's ability to afford healthcare. Some research has indicated that a higher risk of contracting an infectious disease may exist for those living in poverty (63, 64). Through the advanced development of ICTs, the capacity to improve health system efficiencies will be greatly increased and medical errors will be significantly reduced. Thus, our results verify hypothesis 3 that digital economy development mitigates the impact of economic conditions on public health through technological interventions.

## Robustness Checks

### Alternative Variables

To further validate our findings, we conducted robustness tests based on alternative indicators of public health education and digital economy. We used health education training (*edu_train*) as the proxy of public health education, measured by person-time of health education training/population; and used the Peking University Digital Financial Inclusion Index of China (*dfiic*) as the proxy of digital economy development, calculated by Guo et al. (60). The results are shown in columns (1) and (2) in Table 4. *rgdp_train* was negative to *idm* with a coefficient of−0.0042 at a significance level of 5%, thus confirming the moderating effect of public health education. *rgdpg_dfiic* was also significantly negative to *idm* with a coefficient of −0.0036. We therefore conclude that health education and digital economy both alter the effect of business cycle on public health by playing a moderating role.

**Table 4:** Results of robustness checks.

	**Alternative variables**		**System GMM**		
	**(1)**	**(2)**	**(3)**	**(4)**	**(5)**
	***idm***	***idm***	***idm***	***idm***	***idm***
*rgdpg*	0.320^***^	0.558^***^	1.027^**^	0.750^**^	3.552^***^
	(3.96)	(3.98)	(2.55)	(2.12)	(2.92)
*mid*	0.0008	−0.0040	−0.134	−0.0779	−0.225
	(0.05)	(−0.20)	(−1.60)	(−0.78)	(−1.20)
*beds*	0.0127	0.0075	0.138^*^	0.130	0.186
	(1.26)	(0.31)	(1.74)	(1.51)	(1.03)
*doctors*	−0.0342	−0.0323	−0.0008	−0.0911	−0.0254
	(−1.58)	(−1.30)	(−0.01)	(−0.52)	(−0.14)
*s_ind*	−0.0292	−0.246^***^	−0.0028	0.230	−0.0995
	(−0.61)	(−4.22)	(−0.01)	(0.91)	(−0.31)
*ur*	0.0232	−0.749	−1.682	−1.305	−1.518
	(0.11)	(−1.34)	(−1.52)	(−0.67)	(−0.50)
*aging*	1.577^**^	1.080	−1.899	−0.376	4.828
	(2.30)	(0.96)	(−0.51)	(−0.13)	(0.57)
*edu_train*	0.0002				
	(0.61)				
*rgdpg_train*	−0.0042^**^				
	(−2.21)				
*dfiic*		0.0597			
		(1.31)			
*rgdpg_dfiic*		−0.0036^*^			
		(−1.86)			
*edu_act*				0.00214	
				(0.04)	
*rgdpg_act*				−1.214^*^	
				(−1.79)	
*dedi*					−0.821
					(−1.00)
*rgdpg_dedi*					−5.640^*^
					(−1.74)
*L.idm*			−0.334^***^	−0.267^**^	−0.342^**^
			(−3.98)	(−2.13)	(−2.44)
*cons*	0.0636	0.394	1.626^*^	1.112	1.485
	(0.51)	(1.44)	(1.81)	(0.87)	(0.64)
*N*	300	240	270	270	210
*AR(1)*			−2.56	−2.14	−1.72
			(0.011)	(0.033)	(0.086)
*AR(2)*			0.50	1.59	−1.60
			(0.619)	(0.112)	(0.109)
*Hansen*			29.13	28.32	26.18
			(0.849)	(0.874)	(0.794)

*t statistics in parentheses*

* *p < 0.1,*

** *p < 0.05,*

*** *p < 0.01*.

*To save space, the coefficients of control variables are not listed. idm represents infectious disease mortality, rgdpg represents real GDP growth rate, edu_train represents health education training, rgdpg_train is the cross product of rgdpg and edu_train. dfiic represents digital financial inclusion index of China, rgdpg_dfiic is the cross product of rgdpg and dfiic, edu_act represents public health education activity, rgdpg_act is the cross product of rgdpg and edu_act. dedi represents China digital economy development index, rgdpg_dedi is the cross product of rgdpg and dedi, L.idm represents the first-order lag of idm. cons is the constant term, N is the sample observation. AR(1) and AR(2) mean the test statistics of AR(1) and AR(2) models. Hansen means the result of the Hansen test*.

### Alternative Econometric Model

In fact, economy is driven by human capital, which relies on health. There is a potential two-way interaction between business cycle and public health (65). The mortality of infectious diseases may conversely affect the economy. For instance, SARS seriously caused an economic slowdown in China (66). H1N1 and COVID-19 both led to a global economic recession (67–69). Thus, in addition to the fixed-effects model used, we used the dynamic panel system generalized moment estimation method (system GMM), which was proposed by Arellano and Bover (70) and Arellano and Bond (71), to avoid possible heteroscedasticity and autocorrelation in fixed effect estimations. The main advantage of the system GMM over estimators is that it accounts for dynamic panel bias, which arises due to the inclusion of lagged dependent variables. The system GMM is also preferable due to the facts that (i) it fits for empirical growth models with a smaller number of periods and a relatively larger number of countries, (ii) it overcomes the issues of fixed effects and can control endogenous correlation between the first-order lag term of the explained variable and the error term and control possible endogeneity of control variables, and (iii) it yields more consistent and efficient parameter estimates as compared with some other panel data estimators (72). The regression model is constructed as follows:

(4) $$\begin{array}{l} Health_{i,t}=\alpha +\lambda Health_{i,t-1}+\beta {Bus\text{\_}cycle}_{i,t}+\gamma Controls\\ \;+\varepsilon_{it} \end{array}$$

(5) $$\begin{array}{r} Health_{i,t}=\alpha +\lambda Health_{i,t-1}+\beta_{1}{Bus\text{\_}cycle}_{i,t}+\beta_{2}Edu_{i,t}\\ \;+\beta_{3}{Bus\text{\_}cycle}_{i,t}\times Edu_{i,t}+\gamma Controls+\varepsilon_{it} \end{array}$$

(6) $$\begin{array}{r} Health_{i,t}=\alpha +\lambda Health_{i,t-1}+\beta_{1}{Bus\text{\_}cycle}_{i,t}+\beta_{2}Digi_{i,t}\\ \;+\beta_{3}{Bus\text{\_}cycle}_{i,t}\times Digi_{i,t}+\gamma Controls+\varepsilon_{it} \end{array}$$

where *Health*_*i,t*−1_ is the first-order lag term of *Health*_*i,t*_.

According to Blundell and Bond (73) and Guidara et al. (74), the first-order difference of the dependent variable is commonly used as an efficient instrument variable. Thus, we selected the first-order difference of *idm* as the instrumental variable in the system GMM estimation. Furthermore, we used another exogenous factor, i.e., perinatal mortality (*mortality*), as an instrument variable. We collected the data from the China National Health Statistical Yearbook. Columns (3)–(5) in Table 4 show the results estimated by system GMM. The statistics of the AR (2) test and the Sargan test confirmed the validity of selected instrumental variables. The estimated results by system GMM reinforce the fact that business cycle deteriorates public health as the coefficients of *rgdpg* in columns (3)–(5) were all significantly positive. Moreover, public health education and digital economy development alter such harmful effects with the significantly negative coefficients of *rgdp_act* (−1.214) and *rgdpg_dedi* (−5.640), respectively. Thus, the robustness test results show that the conclusions drawn from the previous analysis are indeed robust.

## Conclusions

This paper investigated the relationship between business cycle conditions and public health using a fixed effects model and provided new evidence with respect to previous literature. The results suggested a negative effect of business cycle on public health by increasing the mortality of infectious disease in China. This finding is consistent with the economic stress mechanisms, and similar with the finding of Chai et al. (61). Furthermore, we presented the moderating roles of health education and digital economy development which had been undocumented until now. Empirical results verified the hypothesis that health education and digital economy development buffer the deleterious impact of business cycle on public health. Our findings highlighted the importance of public health education and confirmed that a more educated population will result in a lower mortality of infectious diseases. Health education strengthens the public's health awareness, improves their access to health information, and enhances their capacity to cope with health problems, which helps to mitigate the negative impact of business cycle on public health. The empirical findings of this study also revealed that digitalization has played a crucial role in public health promotion. Digital economy development greatly supports the public health response to health crisis and increases the public's medial accessibility by the application of advanced technologies. It also provides high-quality economic growth, thus avoiding the health issues caused by the traditional economy.

This paper has two implications on public health. For the public, learning more professional knowledge about infectious disease and improving awareness is key to staying healthy. For the government, they should conduct more health education to improve public health literacy. The digital economy, especially digital public health technologies, should be more promoted by the government to mitigate the negative effect of business cycle on public health and thus enhance public health strategies. However, there are still some limitations in the paper, for instance, the potential mechanism underlying how health education and digital economy moderate the effect of business cycle on public health needs to be empirically confirmed in future investigations.

## Data Availability Statement

The raw data supporting the conclusions of this article will be made available by the authors, without undue reservation.

## Author Contributions

XZ: conceptualization, methodology, software, data curation, and writing-original draft preparation. YX: data curation and writing—reviewing and editing. All authors contributed to the article and approved the submitted version.

## Funding

This work was supported by the National Natural Science Foundation of China (Grant Number: 71873014) and the Fundamental Research Funds for the Central Universities, China (Grant Numbers: FRF-BR-20-04B, QNXM20210048).

## Conflict of Interest

The authors declare that the research was conducted in the absence of any commercial or financial relationships that could be construed as a potential conflict of interest.

## Publisher's Note

All claims expressed in this article are solely those of the authors and do not necessarily represent those of their affiliated organizations, or those of the publisher, the editors and the reviewers. Any product that may be evaluated in this article, or claim that may be made by its manufacturer, is not guaranteed or endorsed by the publisher.
